# The impact of reduction in intensity of mechanical ventilation upon venovenous ECMO initiation on radiographically assessed lung edema scores: A retrospective observational study

**DOI:** 10.3389/fmed.2022.1005192

**Published:** 2022-09-20

**Authors:** Elliott T. Worku, Francis Yeung, Chris Anstey, Kiran Shekar

**Affiliations:** ^1^Adult Intensive Care Services, The Prince Charles Hospital, Brisbane, QLD, Australia; ^2^Faculty of Medicine, University of Queensland, Brisbane, QLD, Australia; ^3^School of Medicine, Griffith University, Sunshine Coast Campus, Birtinya, QLD, Australia; ^4^Institute of Health and Biomedical Innovation, Queensland University of Technology, Brisbane, QLD, Australia

**Keywords:** acute respiratory distress syndrome, extracorporeal membrane oxygenation, mechanical ventilation (lung protection) strategy, radiographic assessment scoring systems, RALE

## Abstract

**Background:**

Patients with severe acute respiratory distress syndrome (ARDS) typically receive ultra-protective ventilation after extracorporeal membrane oxygenation (ECMO) is initiated. While the benefit of ECMO appears to derive from supporting “lung rest”, reductions in the intensity of mechanical ventilation, principally tidal volume limitation, may manifest radiologically. This study evaluated the relative changes in radiographic assessment of lung edema (RALE) score upon venovenous ECMO initiation in patients with severe ARDS.

**Methods:**

Digital chest x-rays (CXR) performed at baseline immediately before initiation of ECMO, and at intervals post (median 1.1, 2.1, and 9.6 days) were reviewed in 39 Adult ARDS patients. One hundred fifty-six digital images were scored by two independent, blinded radiologists according to the RALE (Radiographic Assessment of Lung Edema) scoring criteria. Ventilatory data, ECMO parameters and fluid balance were recorded at corresponding time points. Multivariable analysis was performed analyzing the change in RALE score over time relative to baseline.

**Results:**

The RALE score demonstrated excellent inter-rater agreement in this novel application in an ECMO cohort. Mean RALE scores increased from 28 (22–37) at baseline to 35 (26–42) (*p* < 0.001) on D1 of ECMO; increasing RALE was associated with higher baseline APACHE III scores [ß value +0.19 (0.08, 0.30) *p* = 0.001], and greater reductions in tidal volume [ß value −2.08 (−3.07, −1.10) *p* < 0.001] after ECMO initiation. Duration of mechanical ventilation, and ECMO support did not differ between survivors and non-survivors.

**Conclusions:**

The magnitude of reductions in delivered tidal volumes correlated with increasing RALE scores (radiographic worsening) in ARDS patients receiving ECMO. Implications for patient centered outcomes remain unclear. There is a need to define appropriate ventilator settings on venovenous ECMO, counterbalancing the risks vs. benefits of optimal “lung rest” against potential atelectrauma.

## Introduction

ARDS accounts for some 10% of intensive care admissions, and 20% of those requiring invasive ventilation (1), yet our understanding of this heterogenous syndrome and it's management continues to evolve. Venovenous extracorporeal membrane oxygenation, provides life-saving support to patients with severe acute respiratory distress syndrome (2), and recent RCT data suggests that earlier implementation is associated with improved survival (3). Reduced tidal volumes, respiratory rates, and driving pressures are commonly adopted after the commencement of ECMO (4–8), yet consensus is lacking regarding how best to apportion ventilation of the native and membrane lungs, respectively (8, 9). Under ECMO, one may drastically “drop” the native lung, enforcing ultra-protective ventilation (10, 11).

**Figure 1:**
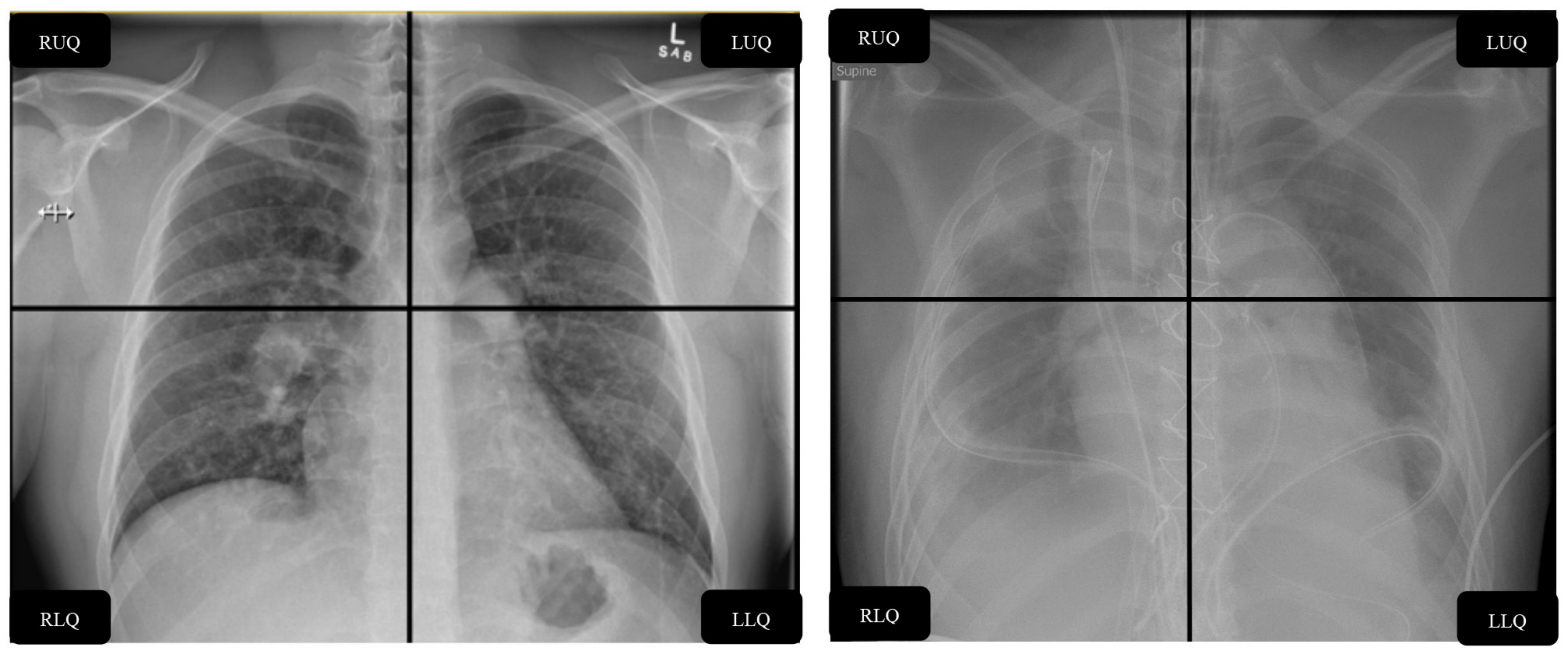
Example calculation of RALE score. RALE, radiographic assessment of lung edema; RUQ, right upper quadrant; RLQ, right lower quadrant; LUQ, left upper quadrant; LLQ, left lower quadrant; CXR, chest X-ray; ECMO, extracorporeal membrane oxygenation.

However, this may engender atelectasis, worsening intrapulmonary shunt (12). Maintenance of adequate extracorporeal blood flows (13) upon which oxygenation will depend when the native lung is “rested”, typically necessitates fluid administration, with increasing fluid balance and extravascular lung water, which may prolong mechanical ventilation (14) and increase mortality on ECMO (15). Increases in lung opacification are anecdotally known to occur shortly after commencing venovenous ECMO (16), and may correlate with mortality based on observational data. The immediacy of this radiological change may result from “dropping the lung” on ECMO (17), exogenous fluid administration, and potential biological injuries inflicted by the extracorporeal circuit (18–20). Bilateral radiographic opacities are intrinsic to the Berlin diagnostic criteria for ARDS diagnosis (21), yet radiological assessment is not objectively standardized. In a study by Rubenfeld et al. there was only moderate agreement achieved in radiological determination of ARDS diagnosis against the predecessor definition of ARDS (kappa 0.55) (22) with as much as a two-fold difference in diagnostic rates as determined (36 vs. 71%) by individual clinicians. In another study, interrater agreement of radiological interpretation was extremely poor between critical care physicians (Kappa 0.05) (23). In the absence of a primary pneumonic cause with florid bilateral consolidation, 13.6% of patients may not even reach a consensus diagnosis of ARDS, yet the mortality in this contended group remains as high as in confirmed ARDS cases (24). The radiographic assessment of lung edema (RALE) score was recently developed to better communicate radiographic changes and has been validated in ARDS cohorts (25), including patients with COVID-19 RDS (26, 27). The score provides a semi-quantitative measure of gravimetric lung edema, and has consistently shown excellent interrater agreement (25, 28), accurately discriminates ARDS from other causes of respiratory failure, and predicts the need for mechanical ventilation arising (27). While the relationship between baseline RALE scores and mortality is inconsistent, there is evidence in COVID-19 that the trend in RALE score in the days following intubation has prognostic relevance (27). As first described by Warren and colleagues (25), the lung fields are divided into quadrants and assigned scores describing the extent and degree of radiological consolidation with a maximum score of 48 (Figure 1). This score has consistently demonstrated excellent interobserver agreement (25, 28), and may improve the utility of CXR, a low cost, and widely available imaging modality that has obvious logistical benefits to CT imaging, especially in light of the unprecedented global pandemic. In this retrospective, single center observational study, we described evolution of radiographic consolidation after the commencement of ECMO. We hypothesized that native lung opacification may be influenced by the degree of lung rest enforced under ECMO, such that RALE scores would increase with reductions in delivered tidal volumes and PEEP, higher fluid balance, and greater ECMO blood flows.

**Table:** 

**Left radiograph (baseline pre-ECMO)**
**Score**	**RUQ**	**RLQ**	**LUQ**	**LLQ**	**Total**
Consolidation	3	3	2.5	3	
Density	1	1.5	1	1	
Quadrant score	3 (3 ×1)	4.5 (3 ×1.5)	2.5 (2.5 ×1)	3 (3 ×1)	13
**Right radiograph (first CXR during ECMO)**
**Score**	**RUQ**	**RLQ**	**LUQ**	**LLQ**	**Total**
Consolidation	3.5	3.5	3.5	4	
Density	3	2.5	2.5	3	
Quadrant score	10.5 (3 ×3.5)	8.75 (3.5 ×2.5)	8.75 (3.5 ×2.5)	12 (4 ×3)	40

## Methods

We performed a retrospective search of patients at our ELSO (Extracorporeal Life Support Organisation) accredited center, to identify all patients with a diagnosis of ARDS (recorded in our electronic admission and clinical information system) who received VVECMO over a 7-year period between January 1st 2012 and December 31st 2019.

**Figure 2:**
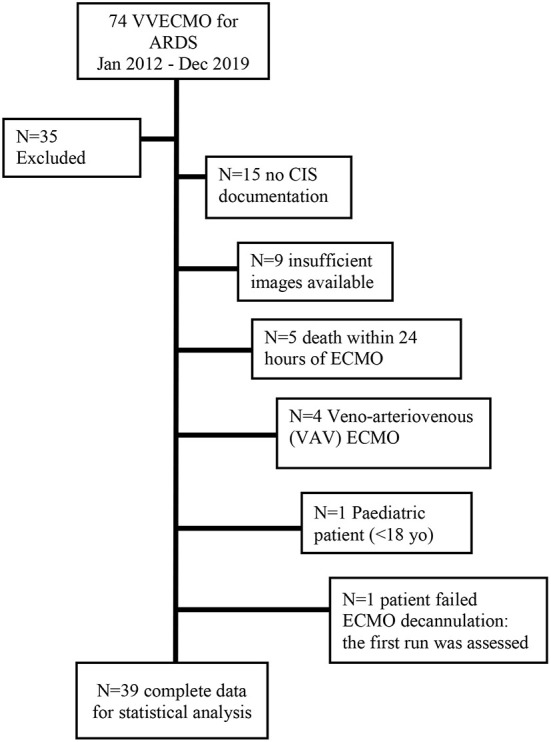
Flowchart for patient inclusion. CIS, clinical information system.

A total of 74 VVECMO runs were conducted for ARDS during the study period, of which 39 individual runs were eligible for inclusion and analysis, satisfying the following criteria: adult patient, >24 h of ECMO, at least 3 digital CXR images available for analysis, and data fully accessible *via* the clinical information system (Figure 2). Demography and illness severity, also retrieved from the electronic record system were documented. De-identified digital chest x-rays were collected from the electronic radiology information system, for patients immediately pre, and post (Median 1.1, 2.1, and 9.6 days) (Supplementary material), initiation of ECMO. These were reviewed by two independent radiologists, blinded to the study hypothesis. The change in lung volumes before and after ECMO were quantified on digitally processed chest radiographs according to the RALE criteria (see below). At each time point, cumulative fluid balance, ventilatory (tidal volume/respiratory frequency/positive end expiratory pressure/FiO_2_) and ECMO parameters: extracorporeal blood flow rates, pump speed, and sweep gas flow and composition were also captured from the clinical information system (Metavision®, iMDSoft, Israel). Ethical approval was sought from The Prince Charles Hospital Human Research Ethics Committee (EC00168), and granted with a waiver of consent (project ID 48432).

### Radiographic scoring—RALE score

The RALE score (25), was calculated as the product of consolidation (0–4) and density scores (1–3) for each of 4 radiographic quadrants (right upper/lower and left upper/lower). The maximum score attainable was 12 per quadrant, and thus 48 in total, which would correspond to dense consolidation occupying > 75% of each radiographic quadrant. Two trained, specialist radiologists: AJW and TR, blinded to the study hypothesis (blinding to ECMO status was not possible due to the presence of radio-apparent cannula) independently scored all deidentified chest radiographs, across all time points (see Figure 1 for example).

### Interobserver agreement for RALES scores

The RALE scores of the two independent reviewers were compared. There was excellent inter-rater agreement as reflected by Cohen's Kappa values of 77.8% (pre-ECMO CXR), 75.5% (day 1 CXR), 83.8% (day 2 CXR), 82.6% (day 6+ CXR). As a result, mean RALE scores were considered acceptable for use in subsequent data analysis.

### Statistics

Summary statistics are presented as number (%) for binary and categorical data, mean (SD) for normally distributed continuous data and median (IQR) for non-normal continuous data. Normality was assessed using the Shapiro-Wilk test. Where appropriate, differences were tested using either a standard *t*-test for normally distributed data or a Wilcoxon rank-sum test for non-normal data. Categorical and binary data were analyzed using Fisher's exact test. ANOVA was used on normally distributed data to test whether the samples originated from the same distribution whereas the Kruskal-Wallis H test was used for non-normal data.

For regression analysis, the data constituted a longitudinal study (panel set) with measurements taken from several patients over time and were analyzed as such with the patients forming the panel series and the day of RALE assessment forming the time element. Regression slope (β) was reported with its 95% confidence interval and *p*-value. Ultimately, model fit was documented using the R^2^ value. Throughout the level of significance was set at α <0.05. STATA™ (version 15.0) was used for all analyses.

### Outcome measures

The primary outcome was the change in RALE score over time, from baseline CXR (the most recent taken immediately prior to ECMO institution), and then at a median of 1.1, 2.1, and 9.6 days post initiation of ECMO therapy (see Supplementary material).

## Results

**Figure 3:**
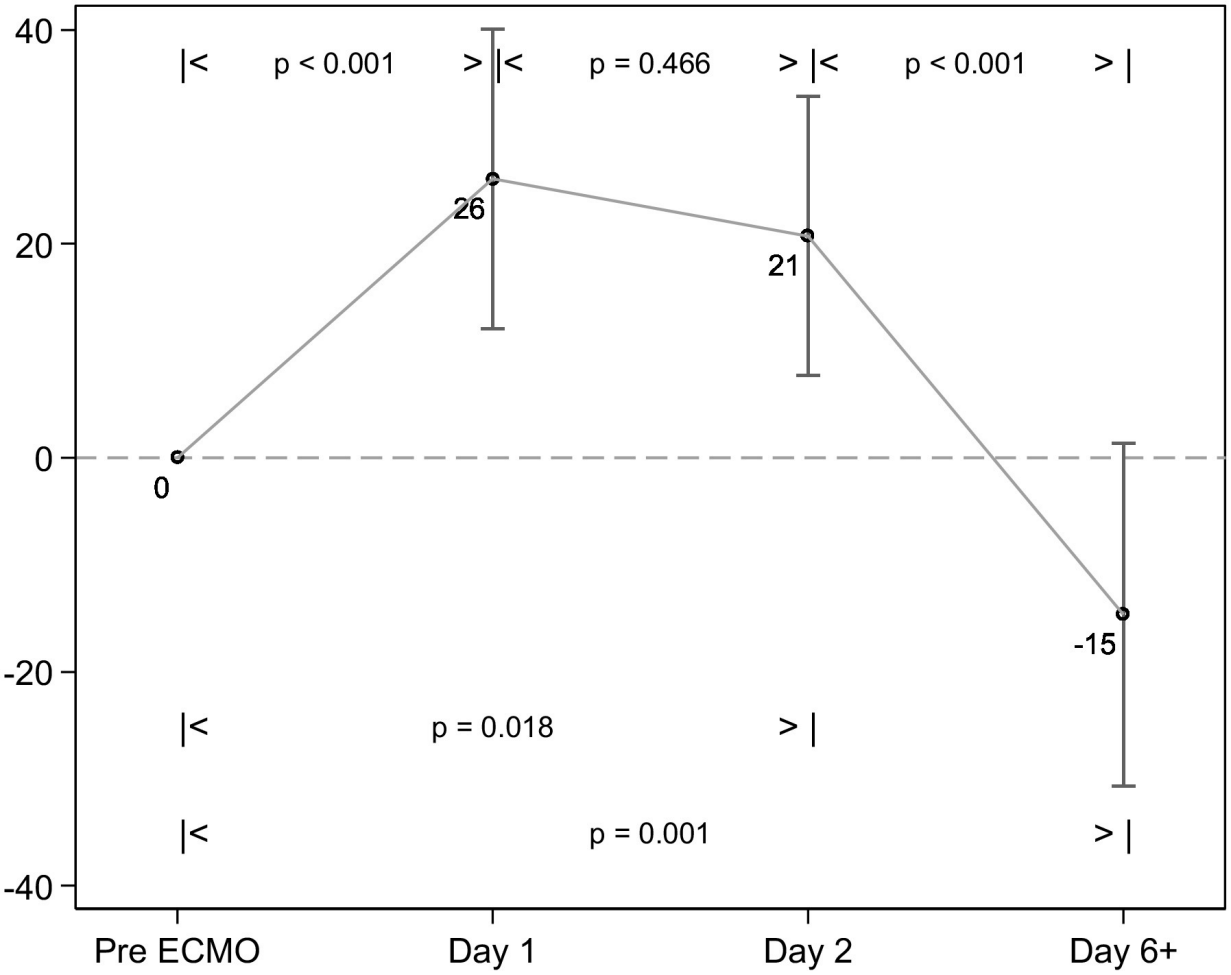
Change in RALE scores over time. The RALE score significantly increased between baseline and 24 h of ECMO therapy. There is a further significant reduction in consolidation seen between ~48 h of ECMO, and the Day 6+ chest X-ray (CXR).

Thirty-nine adult patients (53.9% male), with a median age of 39 years (IQR 30, 57), receiving venovenous ECMO for ARDS were included (Table 1). In total, 6 patients died (15.4% mortality), and median ICU length of stay (LOS) was 548 (279,947) hours, with 428 (165,913), and 212 (113,460) ventilator and ECMO hours, respectively (Table 1). Patient demography, illness severity—as measured by APACHE II, APACHE III, and SOFA (sequential organ failure assessment) scores did not differ significantly between survivors and non-survivors, nor did ICU and hospital LOS or ventilation hours/ECMO duration. With respect to ventilation changes over time, both ventilator FiO_2_ and PEEP decreased from Day 2 onward and tidal volume decreased from baseline to Day 2 then increased thereafter (Tables 2, 3). Absolute RALE scores increased significantly from baseline across the first two time points (*p* = 0.018), with a reduction seen thereafter to below baseline values at the final timepoint (*p* < 0.001) (Table 3; Figure 3). All changes reached statistical significance. Percentage changes in RALE scores compared to baseline values are described in Table 4. Multivariate analysis was performed with mean RALE score as the dependent variable using two models which incorporated demographic, ventilatory and ECMO data (model 1, Supplementary material), and one which omitted ECMO parameters (model 2, Supplementary material). In both models increases in RALE score demonstrated a significant association with increased severity of illness (APACHEIII score) at baseline, and with reductions in tidal volume after the initiation of ECMO. No significant interactions between other ventilator parameters, ECMO variables and RALE score were appreciated.

**Table 1:** Patient demography.

**Variable**	**All data**	**Died**	**Survived**	***p*-value**
*N*	39	6 (15.4%)	33 (84.6%)	–
**Demographics**
Age (yrs)	39 (30, 57)	46 (36, 62)	37 (28, 57)	0.275
Male sex	21 (53.9%)	5 (83.3%)	16 (48.5%)	0.190
Weight (kg)	77 (69, 85)	80 (72, 85)	77 (69, 83)	0.613
**Length of stay (hrs)**
ICU LoS	548 (279, 947)	785 (165, 1,008)	530 (281, 881)	0.117
Hospital LoS	930 (509, 1,164)	795 (177, 1,008)	930 (550, 1,229)	0.199
**Severity of illness**
APA2	21 (18, 26)	23 (17, 29)	21 (18, 25)	0.668
APA3	71 (60, 85)	64 (52, 96)	73 (62, 84)	0.697
APA2 ROD	0.39 (0.29, 0.47)	0.31 (0.24, 0.41)	0.39 (0.30, 0.51)	0.348
APA3 ROD	0.26 (0.15, 0.42)	0.21 (0.08, 0.41)	0.26 (0.16, 0.42)	0.496
SOFA D1	9 (7, 11)	9 (4, 11)	9 (8, 11)	0.481
SOFA D3	8 (6, 10)	9 (4, 12)	8 (6, 9)	0.724
SOFA D5	7 (5, 9)	7 (5, 12)	7 (5, 9)	0.716
**Ventilation and ECMO times (hrs)**
Ventilation	428 (165, 913)	785 (165, 1,006)	424 (180, 855)	0.436
ECMO	212 (113, 460)	638 (165, 885)	205 (113, 353)	0.139

APA, Acute physiology assessment; ROD, Risk of death; SOFA, Sequential organ function assessment; ECMO, Extracorporeal membrane oxygenation.

**Table 2:** Ventilatory and ECMO variables by nominal day.

**Variable**	**Pre ECMO**	**Day 1**	**Day 2**	**Day 6+**
**Ventilatory**
Resp rate (bpm)	28 (20, 30)	14 (10, 19)	15 (10, 23)	23 (20, 28)
Vt (ml)	370 (300, 400)	200 (130, 300)	200 (140, 350)	400 (280, 500)
Vt (ml/kg)	4.5 (4.0, 5.3)	2.5 (1.7, 4.2)	2.5 (1.7, 4.9)	5.6 (3.8, 7.1)
FiO_2_	1.0 (0.75, 1.0)	0.5 (0.4, 0.5)	0.5 (0.4, 0.5)	0.45 (0.4, 0.5)
PS (cmH_2_O)	10 (10, 10)	10 (10, 10)	10 (10, 13)	10 (10, 14)
PEEP (cmH_2_O)	17 (14, 20)	10 (10, 15)	11 (10, 15)	10 (10, 12)
iNO (ppm)	20 (20, 20)	20 (18, 20)	20 (18, 20)	15 (8, 20)
**ECMO**
Flow (lpm)	–	4.8 (3.5, 5.4)	4.7 (3.6, 5.4)	4.0 (3.3, 4.8)
RPM	–	3,340 (2,845, 3,570)	3,388 (2,873, 3,575)	3,030 (2,598, 3,356)
FGF (lpm)	–	6.25 (4.25, 8.00)	6.50 (3.25, 8.00)	0.00 (0.00, 0.75)
FiO_2_	–	1.00 (0.90, 1.00)	0.90 (0.75, 1.00)	0.31 (0.21, 1.00)
CFB (ml)	+297 (−348, +1,583)	+2,197 (+39, +4,020)	+2,585 (+691, +4,525)	+3,111 (−323, +5,979)

Vt, Tidal volume; FiO_2_, fraction of inspired oxygen; PS, Pressure support; PEEP, Positive end-expiratory pressure; iNO, inhaled nitric oxide; RPM, revolutions per minute; FGF, Fresh gas flow (Sweep); CFB, cumulative fluid balance.

**Table 3:** RALE score and ventilation parameters over time.

**Variable**	**Baseline (pre ECMO)**	**Day 1**	**Day 2**	**Day 6+**	***p*-value**
RALE	28 (22, 37)	35 (26, 42)	32 (22, 42)	19 (14, 26)	0.001
FiO_2_	1.00 (0.75, 1.00)	0.50 (0.40, 0.50)	0.50 (0.40, 0.50)	0.45 (0.40, 0.50)	0.001
Vt (ml per kg)	4.5 (4.0, 5.3)	2.5 (1.7, 4.2)	2.5 (1.7, 4.9)	5.6 (3.8, 7.1)	0.001
PEEP	17 (14, 20)	10 (10, 15)	11 (10, 15)	10 (10, 12)	0.001

RALE, Radiological assessment of lung edema; FiO_2_, fraction of inspired oxygen; Vt, Tidal volume; PEEP, Positive end-expiratory pressure.

**Table 4:** Comparison of RALES scores at intervals compared to baseline CXR.

**Day**	**Mean, SE (%)**	***P*-value**	**Median (%)**	**IQR (%)**
1	26 (9)	<0.001	12	0;28
2	21 (9)	0.466	10	−8;33
6+	−15 (11)	<0.001	−30	−57;8

Results described as Mean (SD), with significance described following Wilcoxon rank sum test. The ΔRALE Score was calculated as (Post ECMO – Pre ECMO)/Pre ECMO and expressed as a percentage.

### Model 1: 71 data points R^2^ 0.54

APACHE III: ß value +0.19 (0.08, 0.30) *p* = 0.001

Vt/Kg: ß value −2.08 (−3.07, −1.10) *p* < 0.001

### Model 2: 93 data points R^2^ 0.41

APACHE III: ß value +0.19 (0.09, 0.29) *p* < 0.001

Vt/Kg: ß value −1.53 (−2.28, −0.79) *p* < 0.001

## Discussion

To our knowledge, the current study is the first to report changes in RALE score under the conditions of venovenous ECMO for ARDS. The key finding was a significant increase in radiological edema within 1.1 days of ECMO, independently associated with the severity of illness at admission (APACHE III score), and the magnitude reductions in tidal volumes enforced after ECMO initiation. However, RALE scores were not significantly correlated with ECMO blood flow rate or fluid balance, and while median PEEP was significantly reduced from 17 cm H_2_O to 10 cmH_2_O soon after ECMO initiation, this also failed to associate with increasing radiological opacification. This does not preclude interaction in individual patients, and the initial PEEP levels are higher than previously reported in RALE literature, reflecting the high severity of our study population (25, 28, 29). Despite the initial worsening of RALE score, there was a significant reduction in scores at the final timepoint (median 9.6 days post cannulation) compared to baseline. This may have resulted from resolving ARDS, or pulmonary re-expansion resulting from the significant increase in delivered tidal from median 2.5 ml/Kg (1.7–4.2) immediately post ECMO initiation, to 5.6 ml/Kg (3.8–7.1) at the later time point *p* = 0.001 (Table 3).

All 39 patients can be considered extremely high severity by virtue of requiring ECMO support; the relatively low mortality of 15.4% is suggestive of a mature ECMO service (30). Within our study RALE score did not correlate with mortality; perhaps the discriminatory potential of the score is lost in patients requiring ECMO, or as this study was primarily descriptive, it lacked power to assess these clinical outcomes.

In a prospective study by Kotok et al. reductions in RALE score of >50% by day 7, associated with reduced 90 d mortality, and with early liberation from mechanical ventilation. These findings suggest that the trajectory of radiographic opacification may be prognostically relevant (28). However, RALE score quartiles demonstrated no association with tidal volume, driving pressure or PEEP, and no patients received ECMO, rendering comparison to our cohort unwise. In the initial validation study by Warren et al. a restrictive fluid strategy was associated with a reduction in RALE score over the first 3 days (25), and each 5 point drop in the RALE score corresponded to an 8.4 mmHg increase in the PaO_2_; FiO_2_ ratio (25), suggesting that the score may be used to inform fluid management in ARDS patients and monitor response to diuresis (31). Interestingly, neither cumulative fluid balance nor ECMO blood flow rate appeared to influence the degree of lung edema in our study. Without detailed assessment of individual patient cardiac output, and the corresponding “dose” of ECMO, it is unclear what this means. Previous association of fluid balance during VVECMO and survival shown by Schmidt et al. may reflect patients with greater severity of disease exhibiting higher reliance on ECMO for gas exchange, thus receiving greater fluid loading to sustain the requisite ECMO blood flow (15). ARDS is a heterogenous entity, and dichotomous responses to ventilation (32), prone positioning and fluid management have been demonstrated (33) by etiology, biological profile, radiological morphology, and histological findings (34). While overlap exists (35), extrapulmonary ARDS associates with diffuse radiological abnormalities, and a hyperinflammatory phenotype. This is also associated with higher mortality (36), duration of mechanical ventilation, and elevated markers of sRAGE (37), a biomarker of lung epithelial injury (25, 29). From the median RALE scores reported in our study, one might anticipate more diffuse radiological lung injury, supportive of which RALE score was significantly associated with the baseline APACHE III score; this is speculative without prospective individual patient data. In the LIVE study, Constantin et al. studied individualized ventilation, personalized to the radiological morphology of ARDS, using more liberal tidal volumes and modest PEEP in patients with focal infiltrates (38–41), and emphasizing lower tidal volume and higher PEEP and prone positioning in diffuse ARDS which has a greater potential for recruitment (38, 42–47). Radiological misclassification occurred in a fifth of patients. Where personalized ventilation was correctly aligned with phenotype, individualized care was beneficial, the converse was true with a misaligned ventilation strategy (39). In the same study RALE score values did not correlate with driving pressures, static lung compliance or PEEP (39). In other words, opacification did not alter with reduction in ventilation intensity, thus there is an apparent discrepancy with our findings which may be due to differences in severity of illness, or may suggest that the rapid change in RALE we report are attributable to the ECMO circuit and biological injury from the non-endothelialised circuit (18, 20). Lastly, we must consider the temporal association between ARDS development and clinical outcome. Early onset ARDS may be associated with greater shock severity, larger transfusion burden, and significantly elevated sRAGE (48) suggesting pathophysiological differences from later onset disease. In a recent study, late onset ARDS (>48 h post admission) was a major risk factor for mortality (49). We did not include duration of admission prior to ECMO in our study, thus may have incorporated patients with heterogeneous onset, and varying durations of mechanical ventilation to ECMO. The RALE score has consistently demonstrated excellent interobserver reliability, and so may offer reliable means of diagnosing ARDS (50) and tracking radiological progression. While CT imaging may better inform the proportion of recruitable lung (42, 45, 47, 51), it is logistically less feasible to perform at regular intervals in critically ill patients undergoing ECMO, particularly in the context of a global pandemic.

## Limitations

The current study is limited by reporting a small, retrospective cohort from a single center. ARDS sub-phenotype for individual patients was not reported, thus important interactions between PEEP and RALE score by morphological subtype may have been obscured. Nor was this study powered to assess interaction between the observed radiological changes during ECMO and clinical outcomes. While tidal volume demonstrated an important interaction with RALE score, we cannot infer whether this was mediated by the anticipated driving pressure reductions. Unfortunately, the data required to calculate driving pressures is not included in our local data clinical information system. Fluid management and ventilator strategies on ECMO were not protocolised, and the timing of chest radiographs was not prospectively mandated. During the 7-year span of the study there is likely to have been evolution in oxygen saturation targets, ventilatory management, and transfusion thresholds during ECMO support (3, 6, 52), all of which may influence the degree of radiological opacification. While the interobserver agreement was considered sufficient such that mean RALE scores could be relied upon; higher degrees of correlation have been seen in previous studies (25, 28, 29, 53), perhaps reflecting more formalized radiologist training. Prospective study should attempt to clarify whether the increase in RALE score post ECMO is a phenomenon attributable to the “dose” of ECMO, or due to the aggressiveness of lung protection. Predictive enrichment (54–56) could incorporate radiological morphology and measurement of plasma biomarkers to better address clinical heterogeneity through identification of sub-phenotypes. There is a need to prospectively evaluate various strategies to minimize harm during ECMO, whilst protecting the integrity of native lung function.

## Conclusion

Optimal integration of the membrane and native lungs is elusive. The initiation of ECMO was associated with rapid, and significant increases in radiological consolidation, which correlated with the reductions in delivered tidal volume enforced under ECMO. There is need for external validation of the RALE score in ECMO cohorts to determine its utility in informing clinical management.

## Data availability statement

The raw data supporting the conclusions of this article will be made available by the authors, without undue reservation.

## Ethics statement

The studies involving human participants were reviewed and approved by The Prince Charles Hospital Human Research Ethics Committee (EC00168), and granted with a waiver of consent (project ID 48432). Written informed consent for participation was not required for this study in accordance with the national legislation and the institutional requirements.

## Author contributions

KS and FY conceived the study. FY performed the data collection and ethics application. CA performed all statistical analyses. EW and FY produced the initial draft of the manuscript. EW developed the manuscript and led the revisions process, to which all authors contributed. The final manuscript was approved by all authors.

## Conflict of interest

Author KS is a member of the ECMONet scientific committee, the Asia-Pacific ELSO educational committee, and Australia and New Zealand's Intensive Care Society COVID-19 working group; he is also the lead of an ECMOed research working group and KS acknowledges research support from Metro North Hospital and Health Service. The remaining authors declare that the research was conducted in the absence of any commercial or financial relationships that could be construed as a potential conflict of interest.

## Publisher's note

All claims expressed in this article are solely those of the authors and do not necessarily represent those of their affiliated organizations, or those of the publisher, the editors and the reviewers. Any product that may be evaluated in this article, or claim that may be made by its manufacturer, is not guaranteed or endorsed by the publisher.
