# Supplementary material for: The impact of reduction in intensity of mechanical ventilation upon venovenous ECMO initiation on radiographically assessed lung edema scores: A retrospective observational study
Source: Front Med (Lausanne). 2022 Sep 20;9:1005192. doi: 10.3389/fmed.2022.1005192 (PMC9531725; doi:10.3389/fmed.2022.1005192)
Supplement: Supplementary file 1 [file Data_Sheet_1.docx]

**Supplementary Materials**

Table 1. Timing of Chest Radiographs (From baseline in days)

| Xray time | Median (IQR) | Range | Mean (SD) |
| --- | --- | --- | --- |
| Pre ECMO | - | - | - |
| 1^st^ Post ECMO | 1.1 (0.9, 1.4) | 0.3 to 4.8 | 1.3 (0.7) |
| 2^nd^ Post ECMO | 2.1 (1.9, 2.4) | 1.4 to 5.9 | 2.4 (0.7) |
| 3^rd^ Post ECMO | 9.6 (6.1, 17.7) | 3.2 to 82.8 | 15.0 (15.1) |

Methods: RALE (Radiographic assessment of lung oedema) Score Calculation

| **Consolidation** |  |
| --- | --- |
| Score | Extent of Consolidation |
| 0 | None |
| 1 | <25% |
| 2 | 25-50% |
| 3 | 50-75% |
| 4 | >75% |
| **Density** |  |
| Score | Density of Alveolar Opacities |
| 1 | Hazy |
| 2 | Moderate |
| 3 | Dense |
| **TOTAL SCORE** |  |
| RUQ = (density x consolidation) | LUQ = (density x consolidation) |
| RLQ = (density x consolidation) | LLQ = (density x consolidation) |
| **TOTAL Score = RUQ+RLQ+LUQ+LLQ** |  |

RUQ, right upper quadrant; LUQ, left upper quadrant; RLQ, right lower quadrant; LLQ, left lower quadrant.

Table 2. (Model 1)

Multivariate analysis: Model using baseline, ventilation and ECMO data

Dependent variable was the change in mean RALE score over time.

|  | Initial model | | Final model | |
| --- | --- | --- | --- | --- |
| Variable | β  (95% CI) | p -value | β  (95% CI) | p -value |
| General variables | | | | |
| Age | -0.14  (-0.21, +0.18) | 0.890 | - | - |
| Sex | +3.79  (-1.71, +9.29) | 0.177 | - | - |
| APA3 | +0.20  (+0.84, +0.32) | 0.001 | +0.19  (+0.08, +0.30) | 0.001 |
| Ventilation variables | | | | |
| RR | -0.13  (-0.35, +0.08) | 0.225 | - | - |
| FiO2 | -1.26  (-11.54, +9.02) | 0.810 | - | - |
| Vt/kg | -2.18  (-3.49, -0.86) | 0.001 | -2.08  (-3.07, -1.10) | < 0.001 |
| PS | -0.32  (-1.0, +0.41) | 0.394 | - | - |
| PEEP | =0.38  (-0.54, +1.30) | 0.394 | - | - |
| ECMO variables | | | | |
| Flow | +0.10  (-2.19, +2.38) | 0.934 | - | - |
| CFB (L) | +0.04  (-0.56, +0.63) | 0.907 | - | - |

A total of 71 data points had sufficient data to build the model. Final R^2^ = 0.54

β is the regression coefficient; APA3, APACHE 3 score; RR, respiratory rate; FiO_2_, fraction of inspired oxygen; Vt/kg, tidal volume per kilogram body weight; PS, pressure support; PEEP, positive end-expiratory pressure; CFB, cumulative fluid balance.

Table 3. (Model 2)

Multivariate analysis: Model using baseline and ventilation data

Dependent variable was the change in mean RALE score over time.

Model including baseline data and ventilation data. ECMO parameters were omitted in this model.

|  | Initial model | | Final model | |
| --- | --- | --- | --- | --- |
| Variable | β  (95% CI) | p -value | β  (95% CI) | p -value |
| General variables | | | | |
| Age | 0.00  (-0.17, +0.17) | 0.999 | - | - |
| Sex | +3.35  (-1.72, +8.42) | 0.195 | - | - |
| APA3 | +0.20  (+0.09, +0.31) | < 0.001 | +0.19  (+0.09, +0.29) | < 0.001 |
| Ventilation variables | | | | |
| RR | -0.16  (-0.35, +0.03) | 0.105 | - | - |
| FiO2 | +0.91  (-6.01, +7.83) | 0.796 | - | - |
| Vt/kg | -1.31  (-2.16, -0.46) | 0.002 | -1.53  (-2.28, +-0.79) | < 0.001 |
| PS | -0.15  (-0.84, +0.53) | 0.660 | - | - |
| PEEP | -0.01  (-0.45, +0.44) | 0.975 | - | - |

A total of 93 data points had sufficient data to build the model. Final R^2^ = 0.41

The data were analysed as a longitudinal dataset with the patients forming the panels over time. Significant contributors to increased delta RALE scores over time were increasing APA3 score and declining tidal volume.

β is the regression coefficient; APA3, APACHE 3 score; RR, respiratory rate; FiO_2_, fraction of inspired oxygen; Vt/kg, tidal volume per kilogram body weight; PS, pressure support; PEEP, positive end-expiratory pressure.
